# Vaccination with recombinant *Lactococcus lactis* expressing HA1-IgY Fc fusion protein provides protective mucosal immunity against H9N2 avian influenza virus in chickens

**DOI:** 10.1186/s12985-023-02044-9

**Published:** 2023-04-21

**Authors:** Ruihua Zhang, Tong Xu, Ziping Li, Longfei Li, Chunhong Li, Xinrui Li, Zhiyue Wang, Shaohua Wang, Xuejing Wang, Hongliang Zhang

**Affiliations:** 1 Key Laboratory of Preventive Veterinary Medicine, Department of Veterinary Medicine, Animal Science College, Hebei North University, Zhangjiakou, 075131 Hebei China; 2 The Animal Husbandry and Veterinary Institute of Hebei, Baoding, 071001 Hebei China; 3 Shandong Collaborative Innovation Center for Development of Veterinary Pharmaceuticals, College of Veterinary Medicine, Qingdao Agricultural University, Qingdao, 266109 Shandong China

**Keywords:** H9N2 influenza virus, Immune responses, *Lactococcus lactis*, Oral vaccine, Mucosal immune

## Abstract

**Background:**

H9N2 virus is mainly transmitted through the respiratory mucosal pathway, so mucosal immunity is considered to play a good role in controlling avian influenza infection. It is commonly accepted that no adequate mucosal immunity is achieved by inactivated vaccines, which was widely used to prevent and control avian influenza virus infection. Thus, an improved vaccine to induce both mucosal immunity and systemic immunity is urgently required to control H9N2 avian influenza outbreaks in poultry farms.

**Methods:**

In this study, we constructed a novel *Lactococcus lactis* (*L. lactis*) strain expressing a recombinant fusion protein consisting of the HA1 proteins derived from an endemic H9N2 virus strain and chicken IgY Fc fragment. We evaluated the immunogenicity and protective efficacy of this recombinant *L. lactis* HA1-Fc strain.

**Results:**

Our data demonstrated that chickens immunized with *L. lactis* HA1-Fc strain showed significantly increased levels of serum antibodies, mucosal secretory IgA, T cell-mediated immune responses, and lymphocyte proliferation. Furthermore, following challenge with H9N2 avian influenza virus, chickens immunized with *L. lactis* HA1-Fc strain showed reduced the weight loss, relieved clinical symptoms, and decreased the viral titers and the pathological damage in the lung. Moreover, oropharyngeal and cloacal shedding of the H9N2 influenza virus was detected in chicken immunized with *L. lactis* HA1-Fc after infection, the results showed the titer was low and reduced quickly to reach undetectable levels at 7 days after infection.

**Conclusion:**

Our data showed that the recombinant *L. lactis* HA1-Fc strain could induce protective mucosal and systemic immunity, and this study provides a theoretical basis for improving immune responses to prevent and control H9N2 virus infection.

## Background

Avian influenza virus (AIV) has brought great threat to the health of both poultry and human population worldwide. Avian influenza H9N2 virus (H9N2 AIV) pose a significant economic burden to the commercial poultry industry as they cause signs of mild respiratory illness and reduced egg production [1, 2]. It has been reported that some strains of H9N2 AIV can cause severe clinical symptoms and high mortality [3, 4]. At the same time, co-infection of H9N2 AIV with other pathogens can leads to more serious economic losses [3, 4]. Moreover, H9N2 AIV can directly infect humans and other mammal [5, 6]. The H9N2 AIV has played a significant role in the production of new influenza viruses, such as the H7N9, the H10N8, and H5N6, and these internal genes are mainly derived from the H9N2 AIV [7], which shows the control of influenza virus is very important.

At present, vaccination with the inactivated vaccine is the major tool for the prevention and control of H9N2 AIV in the poultry industry. Although inactivated vaccines are used in the poultry industry to prevent and control influenza, influenza still occurs frequently. That is to say, the inactivated vaccines cannot provide the desired protective effect; the possible reasons could be low mucosal immunity and cellular immunity. H9N2 AIV mainly spreads through respiratory mucosa, so mucosal immunization plays a crucial role in controlling avian influenza infection. Therefore, increasing mucosal or cellular immunity might be pivotal to relieve the limitations of the inactivated vaccine immunization. Given that oral vaccine can induce mucosal immunity and systemic immunity, it is expected to be an effective way to prevent and control H9N2 avian influenza infection.

*L. lactis* is a kind of food-grade lactic acid bacteria, which is considered as an ideal host bacteria for expression of recombinant antigens of pathogenic microorganisms. Actic acid bacteria occurring naturally in the intestines of humans and animals can suppress the reproduction of harmful bacteria, keep the balance of gastrointestinal flora, and improve gastrointestinal function [8]. In addition, Lactic acid bacteria can activates immune cells, improves mucosal immune mechanisms and immune responses [9–11]. It has been reported that Lactic acid bacteria is used as the potential vehicle for protein delivery and induce effective antiviral immune responses, such as rotavirus spike-protein subunit VP8, infectious bursal disease virus VP2 and VP3 [12, 13]. Moreover, *L. lactis* was widely used for mucosal delivery of therapeutic proteins [9, 14]. So the recombinant proteins expressed by *L. lactis* vehicle can induced mucosal and systemic immunity and well protect the challenge animals [15]. So as a safe oral live vaccine delivery carrier, *L. lactis* has a potential application considering its excellent immune effect.

Mammalian IgG molecules can be divided into two Fab fragments, which combine highly variable antigens, and one Fc regions, which recruits and activates immune effector leukocytes. The Fc fragment can bind to its receptor FcR widely expressed on the surface of antigen-presenting cells and mucosal epithelial cells, and the interactions of Fc with activating FcR can activate macrophages, mediate antigen capture, increases the efficiency of the antigen-presenting cells for antigen presentation and triggers effector functions for the immune response [16, 17]; it can also strongly influence the production of cytokine by stimulated macrophages [18]. The most striking feature of macrophage activation is an increase in phagocytic activity, which contributes the antigen presentation to T cells to triggering adaptive immune responses [19]. In avian species, although the Fc segments of IgY is different in structure structures, IgY is similar to mammalian IgG in terms of functionality [20, 21]. It has been reported that the linked IgY Fc mediated the interaction with macrophages and increases the efficiency of antigen-processing, thereby improving the immune response induced by the antigen [20].

The neonatal Fc receptor (FcRn) of IgG is expressed on the surfaces of antigen presenting cells and mucosal epithelial cells in adulthood. FcRn can transport maternal IgG to the newborn before the immune system of the newborn matures, so that the newborn can acquire immune defense against pathogens to resist external diseases [22]. Numerous reports have confirmed that targeting protective antigens to FcRn can increased humoral and cellular immune responses [23–25]. In avian species, the Fc receptor with the requisite properties for IgY transport from yolk to embryo, named FcRY, were isolated and characterized from chicken yolk sac [26]. It was reported that besides the yolk sac membrane, FcRY expression was also observed in most tissues, incluing liver, ovary, oviduct, ileum, and spleen [26]. This may reflect the role of IgY is similar to FcRn’s function to IgG in mammals [27, 28]. So, in our study, we targeted protective antigens of H9N2 AIV to FcRY to explore whether the linked chicken IgY Fc fragment fusion could increase immune responses.

Hemagglutinin is the main surface antigen of influenza A virus and the primary target for the production of specific neutralizing antibodies [29–31]. So HA antibody is particularly important in the fight against infection and disease and is a crucial target for vaccine development. HA is a homotrimer, each monomer is synthesized as a single polypeptide (HA0) that is split into two HA1 and HA2 subunits by matriptase in host cells [32, 33]. The N-terminal of HA1 forms a globular head structural domain; the HA1 domain contains many antigenic determinants that stimulate the production and binding of neutralizing antibodies. HA2 forms a stem structure; HA2 domain anchored to the viral envelope can fuses the virus envelope with the cell membrane and release the nucleocapsid of the virus particle [34]. In a word, the sequence of HA1 can stimulate neutralizing antibodies production; therefore, in this study the oral vaccine based on the region of HA1 is being considered.

In this study, we used the *L. lactis* MG1363 to express a fusion protein containing the chicken IgY Fc and HA1 of H9N2 AIV. The immunogenicity of this recombinant *L. lactis* strain was then evaluated. The protective effect of this recombinant *L. lactis* strain against H9N2 AIV challenge following oral immunization was also evaluated aiming to explore a complementary method for the prevention and control of H9N2 subtype AIV.

## Materials and methods

### Bacteria, virus, and inactivated vaccines

The H9N2 influenza A/pigeon/Hebei/02/2017 virus (H9N2 virus) strain was isolated (GenBank accession numbers Mk995886-Mk995893) from the intestinal tract of a pigeon in Hebei Province and stored in Hebei North University. *L. lactis* MG1363-pMG36e (*L. lactis*), *L. lactis* MG1363-pMG36e-HA1 (*L. lactis* HA1) was stored and prepared as a vaccine candidate from a *L. lactis* MG1363 system in Hebei North University. For inactivated H9N2 vaccine production, A/pigeon/Hebei/02/2017 virus was propagated in the allantoic cavities of 10-day-old SPF embryonated eggs. Allantoic fluid was harvested 72 h after inoculation and treated with formalin (final concentration of 0.2%) for 24 h at room temperature. Then the allantoic fluid was emulsified in Montanide ISA70 (SEPPIC) at a ratio of 30:70 (v/v).

### Construction of recombinant *L. lactis*

To recombine HA1 of A/pigeon/Hebei/02/2017 virus fused to IgY Fc at the N-terminus, the recombinant plasmids pMG36e-HA1-Fc was constructed, and the HA1 gene and the IgY Fc gene were connected by a fifteen-amino acid linker (3XGGGGS). The IgY Fc fragment of chicken was obtained from GenBank (X07174.1). With HA1-Fc gene for the selected target gene, *Sal*I and *Hind*III restriction enzyme cutting sites were added and were chemically synthesized by Gene Wiz, Inc. (Suzhou, China). Then the HA1-Fc gene was cloned into pMG36e vector by digestion with the *Sal*I and *Hind*III restriction sites to create pMG36e-HA1-Fc. Subsequently, the recombinant plasmids were transformed into *L. lactis* MG1363 by electroporation, then the clones were sequenced in Gene Wiz, Inc. (Suzhou, China), and the confirmed positive recombinant *L. lactis* MG1363 was named *L. lactis* MG1363-pMG36e-HA1-Fc(*L. lactis* HA1-Fc).

### Western blotting analysis

To examine HA1-Fc expression, the recombinant *L. lactis* and *L. lactis* HA1-Fc were cultured in MRS medium supplemented with 10 μg/ml erythromycin. When an OD_600_ = 0.8 was reached, the bacteria were harvested by centrifugation and resuspended in TBS buffer, and then were disrupted with sonication. The cell lysate was used to detect the presence of recombinant protein. Proteins were separated by SDS-PAGE and transferred to PVDF membranes. The presence of the fusion protein was detected using rabbit polyclonal to Avian Influenza A H9N2 Hemagglutinin antibody (Novus Biologicals) followed by horseradish peroxidase-labeled goat anti-rabbit IgG (abcam). Visualization of the immunobinding was conducted by enhanced chemiluminescence Western blotting detection system (Amersham Pharmacia Biotech).

### Vaccination and challenge

One-day-old specific-pathogen-free chickens were randomly divided into five treatment groups of 25 chickens each, PBS group, *L. lactis* group, *L. lactis* HA1 group, *L. lactis* HA1-Fc group, H9N2 inactivated vaccine group. PBS group of chickens were orally immunized with PBS (0.2 mL/chicken). *L. lactis* group, *L. lactis* HA1 group, *L. lactis* HA1-Fc group of chickens were orally immunized with 10^9^ cfu/mL of the *L. lactis* MG1363-pMG36e, *L. lactis* MG1363-pMG36e-HA1 and *L. lactis* MG1363-pMG36e-HA1-Fc(0.2 mL/chicken), respectively. H9N2 inactivated vaccine group of chickens received intramuscular injections of H9N2 inactivated vaccine (0.2 mL/chicken). Chickens in all groups except the inactivated vaccine group received a first vaccination at 1, 2, and 3 days and a booster vaccination at 14, 15 and 16 days. Chickens in the inactivated vaccine group were immunized on the first day. Then sera, BALF, spleen, intestinal samples, and feces were collected from immunized chickens on 10 days after the first and boosting vaccination for antibody assays, cytokine detection and lymphocyte proliferation assays. Then, all groups of chickens were intranasally challenged with 10^6.0^ EID_50_ of A/pigeon/Hebei/02/2017 AIV in 0.1 ml of PBS on day 24, and clinical signs, the body weight, protection rates, viral shedding, pulmonary pathological changes and lung tissue virus titers of each group were analyzed. The challenge test with live H9N2 virus was done in the poultry isolators with Hepa Filter.

### HI assay

Sera were collected from all groups of chickens at 10 days after first and boosting vaccination. Hemagglutination Inhibition (HI) antibody titers of serum antibodies were determined following the previous study [35]. The highest serum dilution capable of preventing hemagglutination was scored as the HI-titer.

### Mucosal SIgA and serum IgG antibodies assays

Sera, BALF and feces samples were collected from all groups of chickens at 10 days after first and boosting vaccination. The sIgA antibody levels were measured using the Chicken sIgA ELISA Kit (Lengton Bioscience Co., LTD, Shanghai, China), according to the manufacturer’s protocol for BALF and feces samples. Serum-specific IgG antibody levels were detected by the indirect ELISA method, according to a previously reported method [36]. Briefly, 96 well plates were coated with 1 mg/ml of synthetic HA1-peptide (23 amino acids, 2–26, KICIGYQSTNSTETVDTLTENNVPV, synthesized by Sangon Biotech (Shanghai) Co., Ltd, China) specificity H9N2 subtype AIV HA1 protein, in 50 mM sodium bicarbonate buffer, and incubated overnight at 4 °C. After blocking, serum samples were loaded on peptide coated plates and incubated for 1 h at 37 °C. Subsequently, plates were washed and incubated with goat anti-chicken IgG-HRP conjugates (SigmaAldrich, USA) for another 1 h at 37 °C. The color reaction was developed with TMB, and absorbances were read at OD 450 nm. The optical density represented total mucosal sIgA and serum IgG specific for HA1-Fc. For comparison between groups, the averages of A450 values of different sera were analyzed [37].

### Real-time PCR assays for detection of intestinal mucosal cytokines

IL-2, IL-4, and IFN-γ in Intestinal samples were measured by real-time PCR. Total RNA was extracted using Trizol reagent (Invitrogen). cDNA was synthesized from RNA with cDNA Reverse Transcription Kit (Applied Biosystems). PCR amplification assays were performed with a SYBR Premix Ex Taq II kit (TaKaRa) on an ABI 7300 Real-Time PCR system (Applied Biosystems). The target gene expression was normalized on the basis of β-Actin expression. The 2^−ΔΔCT^ method was used to normalize the data. The experimental primers were listed in Table 1 [15, 38].

**Table 1:** The primers used in this study

Primer	Sense (5′-3′)	Anti-sense (5′-3′)
IL-2	CTCGGAGCTCTGCAGCGTGT	TCCACCACAGTTGCTGGCTCATC
IL-4	CCACGGAGAACGAGCTCATC	GAGAACCCCAGACTTGTTCTTCA
IFN-γ	ACACTGACAAGTCAAAGCCGC	AGTCGTTCATCGGGAGCTTG
β-Actin	CAACACAGTGCTGTCTGGTGGTA	ATCGTACTCCTGCTTGCTGATCC

### Proliferation assay

To detect splenic lymphocyte proliferation of vaccinated chickens, lymphocytes were seeded in 96-well plates at 2 × 10^5^ cells/ml, the cultures were stimulated for 48 h with 5 μg/ml of phytohemagglutinin (PHA, Sigma) as positive control, 5 μg/ml of synthetic HA1 peptide as specific antigen, or RPMI 1640 as negative control, and proliferation rate of the splenic lymphocyte was detected by Cell Proliferation ELISA BrdU Kit (Roche, Germany), according to manufacturer’s protocol. Absorbances were read at 450 nm. Results were expressed as a stimulation index (SI), which was described as the ratio of the average OD of antigen-stimulated cells to the average OD of non-stimulated cells, and the stimulation index is used to express a proliferative response against synthetic HA1 peptide of splenic lymphocyte.

### Analysis of protective immune responses

Challenged chickens were monitored for clinical symptoms and mortality, and changes in body weights were recorded. To detect viral shedding, cloacal and oropharyngeal swabs were collected from challenged chickens on days 2, 4, and 7 post-challenges and suspended in 1 mL of PBS. These time points represent the peak for oropharyngeal and cloacal shedding [39, 40]. Moreover, five randomly-selected chickens from each group were sacrificed and lung tissues were collected, on days 7 post-challenges. The viral titers in the lungs were measured by EID^50^. Pulmonary pathological changes was visualized by hematoxylin and eosin staining.

### Statistical analysis

Statistical analysis was performe International Immunopharmacology d with the SPSS statistical software package for Windows, version 18.0 (SPSS, Inc., Chicago, IL, USA). All data are presented as the means ± SD. Statistically significant differences among groups were calculated by one-way ANOVA followed by Tukey’s multiple comparisons test. *P* < 0.05 was considered significant.

## Results

### Identification of recombinant proteins

**Fig. 1:**
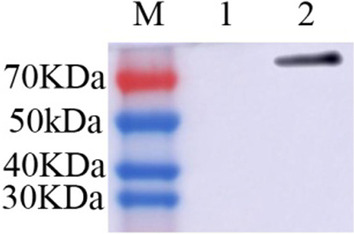
Western blot analyses of *L. lactis* HA1-Fc. Western blotting identification of the recombinant proteins with the goat anti-rabbit HA antibody. Lane M is protein molecular size page ruler; Lane 1 is the strain of *L. lactis*; Lane 2 is the strain of *L. lactis* HA1-Fc

HA1-Fc gene was cloned into the expression vector pMG36e and verified by sequencing, and the recombinant plasmids pMG36e-HA1-Fc was transformed into *L. lactis* MG1363 by electroporation. The expressed protein bands corresponding to 74 kDa in the positive recombinant *L. lactis* HA1-Fc were observed through western blotting. The result confirmed the expression of the recombinant HA1-Fc fusion proteins and the good reactogenicity to the Avian Influenza A Hemagglutinin antibody (Fig. 1).

### HI antibody levels induced by *L. lactis *HA1-Fc

**Fig. 2:**
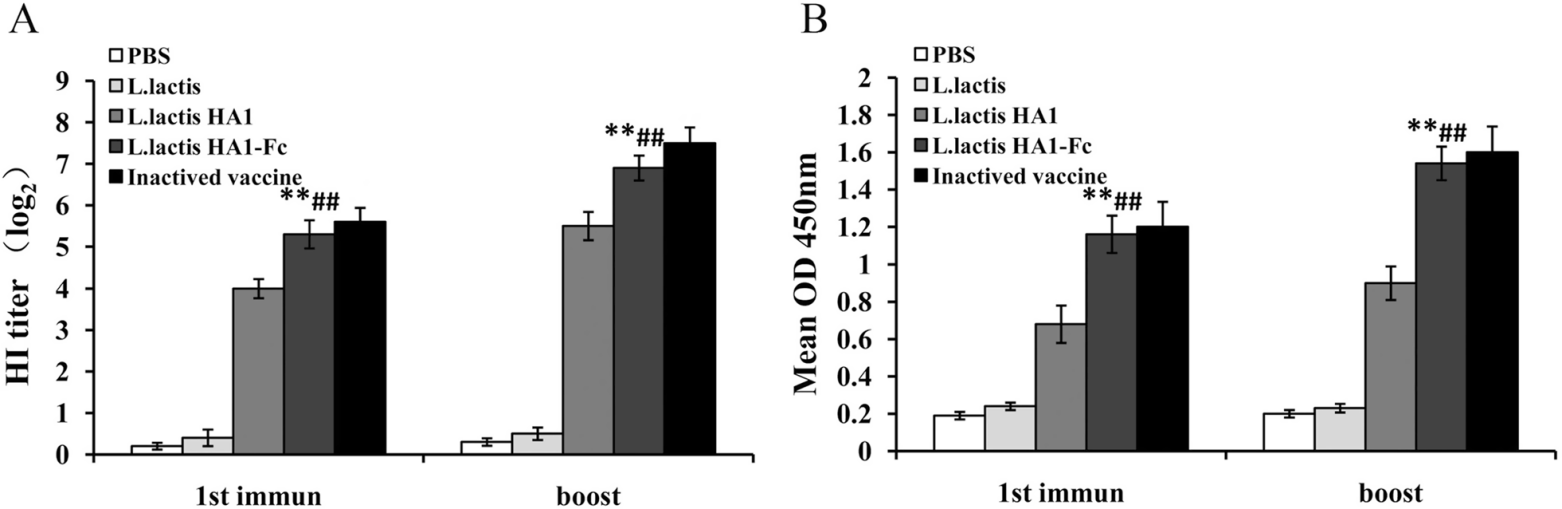
HI Antibody levels and the specific IgG levels induced by *L. lactis* HA1-Fc. **A** Antibody titers determined by HI assay after the chickens were vaccinated with PBS, *L. lactis*, *L. lactis* HA1, *L. lactis* HA1-Fc and inactivated H9N2 vaccine. **B** The antigen specific IgG titers of chickens after vaccination detected by indirect ELISA. The data were showed as means ± SD (n = 5). **P < 0.01 relative to PBS and *L. lactis*. ^##^*P* < 0.01 relative to *L. lactis* HA1

The recombinant *L. lactis* HA1-Fc was used as an oral vaccine to immunize SPF chickens. Then HI antibody levels were measured to determine whether the *L. lactis* HA1-Fc could stimulate the body to produce specific antibodies. The result showed that chickens orally vaccinated with *L. lactis* HA1-Fc had obviously higher HI antibody levels than the chickens that were orally immunized with *L. lactis* or PBS (*P* < 0.01). Moreover, HI antibody levels in chickens orally immunized with *L. lactis* HA1-Fc increased significantly compared with chickens immunized with *L. lactis* HA1 (*P* < 0.01). Our data also showed that although vaccination with the inactivated vaccine induced high HI antibody, no significant differences in HI antibody were detected in chickens immunized with inactivated vaccine and *L. lactis* HA1-Fc (Fig. 2A). This result proved that recombinant *L. lactis* HA1-Fc can stimulate the body to produce specific antibodies and Fc fragment plays an important role in the production of specific antibodies (Fig. 2A).

### The specific IgG Levels enhanced by *L. lactis *HA1-Fc

To investigate the specific IgG levels in chickens after oral vaccination with *L. lactis* HA1-Fc, the serum samples from various groups of chickens were detected by indirect ELISA. As shown in Fig. 2B, compared with the *L. lactis* group and PBS group, the *L. lactis* HA1-Fc group had a much higher specific IgG levels after oral immunization (*P* < 0.001). Notably, the specific IgG titers in the serum from chickens orally vaccinated with *L. lactis* HA1-Fc was increased obviously compared with the *L. lactis* HA1 group (*P* < 0.01). As with HI antibody levels, there was no obvious difference in specific IgG titers between the *L. lactis* HA1-Fc group and the inactivated vaccine group (*P* < 0.01). Therefore, our data can infer that *L. lactis* HA1-Fc is capable of stimulating a strong humoral immune response in chickens.

### SIgA antibodies in the feces and BALF following vaccination

**Fig. 3:**
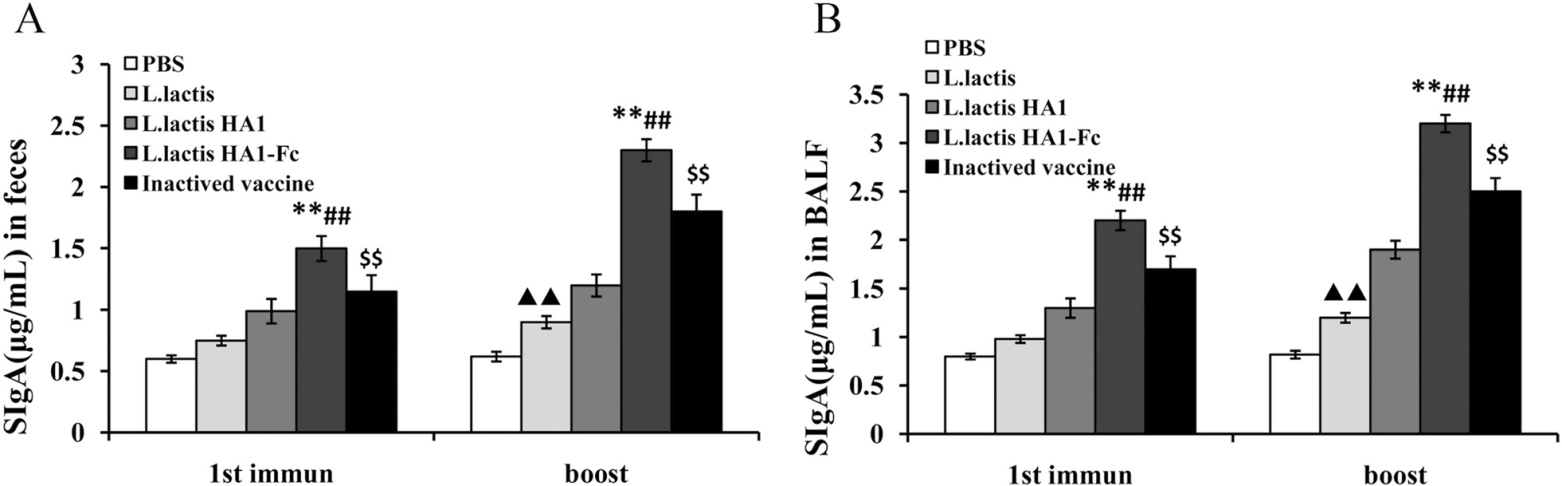
Mucosal antibody levels induced by *L. lactis* HA1-Fc. SIgA antibodies in the feces (**A**) and BALF (**B**) were assessed by ELISA after the chickens were vaccinated with PBS, *L. lactis*, *L. lactis* HA1, *L. lactis* HA1-Fc and inactivated H9N2 vaccine. The data were showed as means ± SD (n = 5). ^▲▲^*P* < 0.01 relative to PBS. **P < 0.01 relative to PBS and *L. lactis*. ^##^*P* < 0.01 relative to *L. lactis* HA1. ^$$^*P* < 0.01 relative to *L. lactis* HA1-Fc

To evaluate whether recombinant *L. lactis* HA1-Fc can induce body mucosal immunity, sIgA antibodies of all groups of chicken was measured after each immunization (Fig. 3). The results showed that intestinal sIgA levels in the *L. lactis* HA1-Fc vaccinated group were higher obviously than those of the PBS vaccinated group and *L. lactis* vaccinated group (*P* < 0.01), and chicken immunized with *L. lactis* HA1-Fc showed higher titer of sIgA antibodies compared with chickens immunized with *L. lactis* HA1 (*P* < 0.01) (Fig. 3A). Moreover, sIgA levels in the *L. lactis* HA1-Fc vaccinated group were also significantly higher compared with the inactivated vaccine group (*P* < 0.01) (Fig. 3A). SIgA levels in BALF showed a similar trend to intestinal sIgA levels (Fig. 3B). SIgA levels in the *L. lactis* HA1-Fc vaccinated group were significantly higher than those in the PBS vaccinated and *L. lactis* vaccinated groups (*P* < 0.01), and chicken immunized with *L. lactis* HA1-Fc showed higher titer of sIgA antibodies compared with chickens immunized with *L. lactis* HA1 and inactivated vaccine (*P* < 0.01). Moreover, there was statistically significant difference in the induced sIgA antibodies in the feces and BALF samples between *L. lactis* and PBS immunized groups of chicken after immunization. Therefore, *Lactococcus lactis* can improves mucosal immune mechanisms and is a good vehicle for protein delivery. *L. lactis* HA1-Fc could induce enhanced mucosal response.

### Intestinal mucosal cytokines induced by *L. lactis *HA1-Fc

**Fig. 4:**
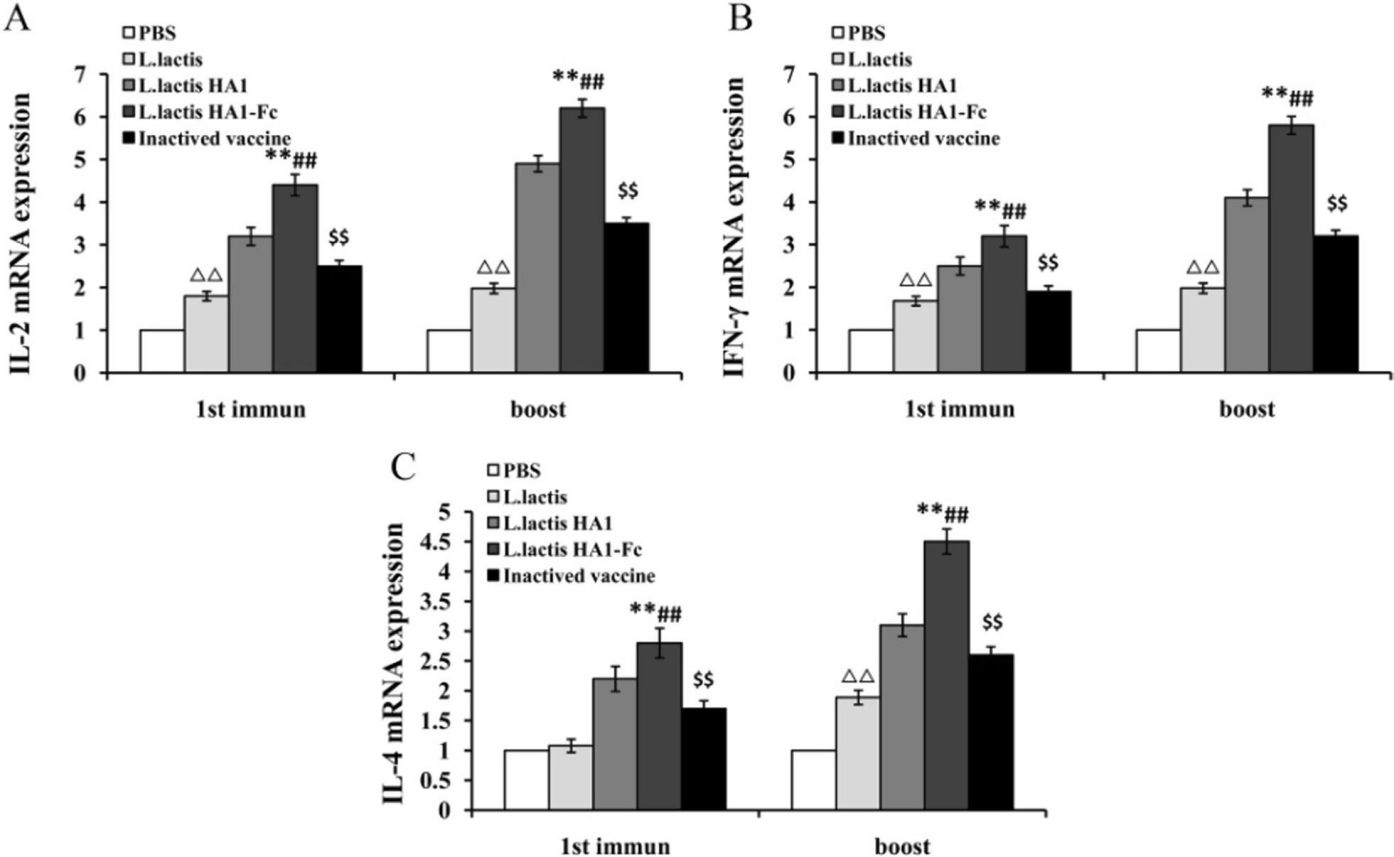
IL-2, IL-4, and IFN-γ levels in intestinal tissues induced by *L. lactis* HA1-Fc. Chickens were vaccinated with PBS, *L. lactis*, *L. lactis* HA1, *L. lactis* HA1-Fc and inactivated H9N2 vaccine, respectively, then intestinal tissues were collected. IL-2 (**A**), IFN-γ (**B**), and IL-4 (**C**) were detected via real-time PCR. The data were showed as means ± SD (n = 5). ^ΔΔ^P < 0.01 relative to PBS. **P < 0.01 relative to PBS and *L. lactis*. ^##^*P* < 0.01 relative to *L. lactis* HA1. ^$$^*P* < 0.01 relative to *L. lactis* HA1-Fc

Cytokines are crucial for fighting off infections and are involved in immune responses, IL-2 and INF-γ primarily stimulate cell-mediated immune response, and IL-4 primarily stimulate antibody production and mediates humoral immune responses [21]. Therefore, in this study intestinal tissues were collected from all chickens and mucosal cytokines levels were measured by real-time PCR. As shown in Fig. 4, levels of IL-2, IFN-γ and IL-4 (as a marker of Th1 and Th2, respectively) in the *L. lactis* HA1-Fc vaccinated group were significantly higher than those in the *L. lactis* vaccinated group and PBS vaccinated group, and the *L. lactis* HA1-Fc vaccinated group showed significantly increased levels of all three cytokines compared with the *L. lactis* HA1 vaccinated groups (*P* < 0.01). There were higher levels of cytokines of IL-2, IL-4 and IFN-γ in the inactivated vaccine groups, but chickens immunized with *L. lactis* HA1-Fc showed higher levels of IL-2, IL-4 and IFN-γ than those immunized with inactivated vaccine (*P* < 0.05). Moreover, there was statistically significant difference in the induced mucosal cytokines levels between *L. lactis* and *PBS* immunized groups of chicken after immunization (Fig. 4). Therefore, the results indicated *L. lactis* HA1-Fc fragment significantly promoted the secretion of cytokines in chickens. *Lactococcus lactis* can activate immune cells and improves immune responses [9–11]. The levels of IL-2 and IFN-γ in chicken immunized with *L. lactis* HA1-Fc were greater than IL-4 levels, which showed the greater Th1-type than Th2-type immune response in chicken.

### Proliferation assay

The cellular immune response was examined by Cell Proliferation ELISA BrdU Kit.

**Fig. 5:**
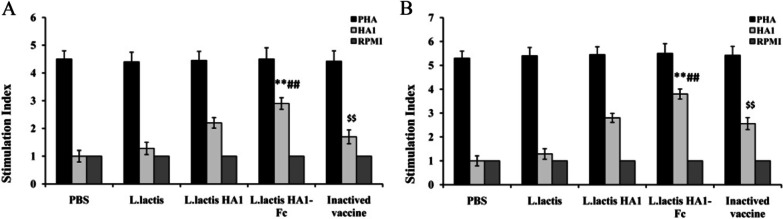
T cells proliferation assay. T cells proliferation was examined by Cell Proliferation ELISA BrdU Kit, and results were expressed as a stimulation index (SI) of chickens vaccinated with PBS, *L. lactis*, *L. lactis* HA1, *L. lactis* HA1-Fc and inactivated H9N2 vaccine. The data were showed as means ± SD (n = 5). **P < 0.01 relative to PBS and *L. lactis*. ^##^*P* < 0.01 relative to *L. lactis* HA1. ^$$^*P* < 0.01 relative to *L. lactis* HA1-Fc

The results showed an enhanced T-cell proliferative response to HA1 peptide was observed in the groups vaccinated with *L. lactis* HA1, *L. lactis* HA1-Fc or inactivated vaccine when stimulated with synthetic HA1 peptide, whereas the chickens immunized with PHA or RPMI did not respond to the HA1 peptide (Fig. 5). Stimulation index in *L. lactis* HA1-Fc group was significantly higher than those in groups *L. lactis* and PBS group. Notably, the group *L. lactis* HA1-Fc showed significantly higher stimulation index than group *L. lactis* HA1 after the first immunization and boost immunization (*P* < 0.05). Moreover, there were higher stimulation index in the inactivated vaccine groups, but chickens immunized with *L. lactis* HA1-Fc showed higher stimulation index than chicken immunized with inactivated vaccine (*P* < 0.05). These results suggested that *L. lactis* HA1-Fc significantly promoted immune responses in chicken.

### Protective effect of *L. lactis *HA1-Fc on H9N2 subtype of AIV

To evaluate the protective effect of recombinant *L. lactis* HA1-Fc strain on the H9N2 subtype AIV, clinical signs, the body weight, protection rates, viral shedding, pulmonary pathological changes and lung tissue virus titers of each group were statistically analyzed after the challenge experiment. From day 3 after viral challenges, all the challenged, PBS and *L. lactis* vaccinated chicken exhibited apparent clinical signs including: depression, poor appetite, ruffled feathers, respiratory sounds, and eye redness from 2 to 8 days post-challenge. About half of chickens in group *L. lactis* HA1 showed clinical signs, and three-quarters of chickens in group *L. lactis* HA1-Fc showed no clinical symptoms. Moreover, the clinical signs were delayed for 1–2 days in the chicken vaccinated with the *L. lactis* HA1-Fc and *L. lactis* HA1, in which the chicken showed mild clinical signs for 2–3 days, and the clinical signs in the chicken vaccinated with the *L. lactis* HA1-Fc were milder than those vaccinated with the *L. lactis* HA1. There was no significant difference in clinical signs between chicken immunized with *L. lactis* HA1-Fc or inactivated vaccine. By day 5 post-challenge, the chickens immunized with *L. lactis* HA1-Fc showed some recovery. On day 7 post-challenge, chickens were behaving normally and their activity and appetite returned to normal levels.

**Fig. 6:**
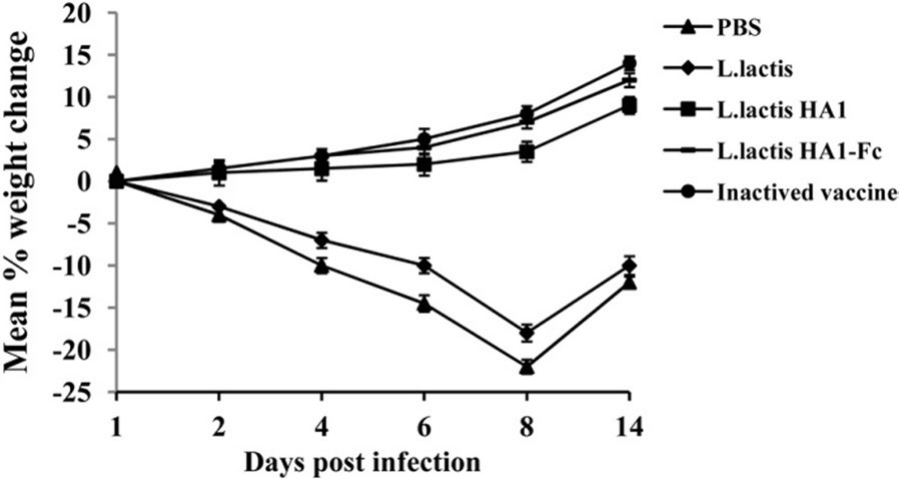
Weight change of the vaccinated chickens after challenge with H9N2 avian influenza virus. Chickens were immunized with PBS, *L. lactis*, *L. lactis* HA1, *L. lactis* HA1-Fc and inactivated H9N2 vaccine. 10 days after the boost immunization, the immunized chickens were challenged with H9N2 influenza virus. The weight loss was recorded daily for 2 weeks

The body weights of the *L. lactis* group and PBS group exhibited downward trends after H9N2 virus infection, and both were obviously lower than the other three groups (*P* < 0.01) (Fig. 6). The chicken in *L. lactis* HA1-Fc group showed less weight loss than that observed for the chicken in *L. lactis* HA1 group, but the weight changes between these two groups were not statistically significant. Moreover, there was no significant difference between the *L. lactis* HA1-Fc group and the inactivated vaccines group (*P* > 0.05).

None of the chickens immunized with *L. lactis* HA1-Fc or inactivated vaccines died after infection with H9N2 avian influenza virus, whereas five of the 25 chickens immunized with PBS and *L. lactis* and two of the 25 chickens immunized with *L. lactis* HA1 died from the disease.

**Fig. 7:**
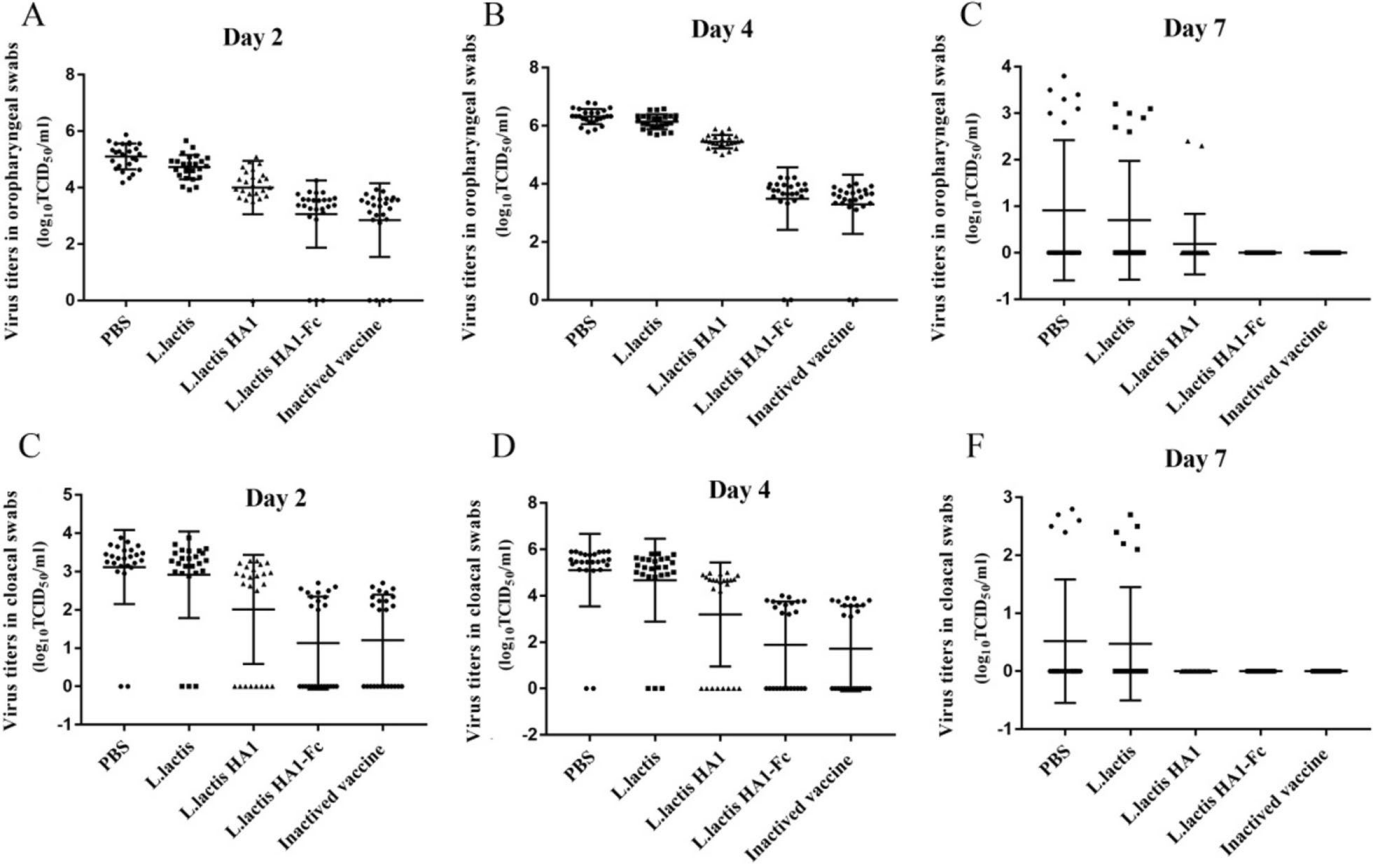
Viral shedding from cloacal and oropharyngeal swabs. Immunized chickens were infected with H9N2 avian influenza virus, cloacal and oropharyngeal swabs were collected from challenged chickens on days 2, 4, and 7 post-challenges. Viral titers were detected from oropharyngeal swabs collected from challenged chickens on days 2 (**A**), 4 (**B**), and 7 (**C**) post-challenges. Viral titers were detected from cloacal swabs collected from challenged chickens on days 2 (**D**), 4 (**E**), and 7 (**F**) post-challenges

To investigate virus shedding, oropharyngeal and cloacal swabs were collected from all chickens on days 2, 4 and 7 after infection with H9N2 avian influenza virus, and virus titration was measured in MDCK cells. The result showed that all chicken in PBS, *L. lactis and L. lactis* HA1 groups showed respiratory viral replication, and several chicken in *L. lactis* HA1-Fc groups did not showed respiratory viral replication at days 2, and 4 post-challenges. The chicken vaccinated with *L. lactis* HA1-Fc had overall significantly lower oropharyngeal and cloacal viral shedding than did the challenged PBS, *L. lactis* or *L. lactis* HA1 immunized chickens (*P* < 0.01). At day 7 post-challenge, no viruses were isolated from oropharyngeal swabs of the chicken vaccinated with *L. lactis* HA1-Fc and inactive vaccine; whereas, viruses were isolated from oropharyngeal swabs of the other infected chickes. As far as the periods of viral shedding was concerned, the chicken vaccinated with *L. lactis* HA1-Fc had lower periods of virus replication (Fig. 7).

### Histopathologic evaluation

**Fig. 8:**
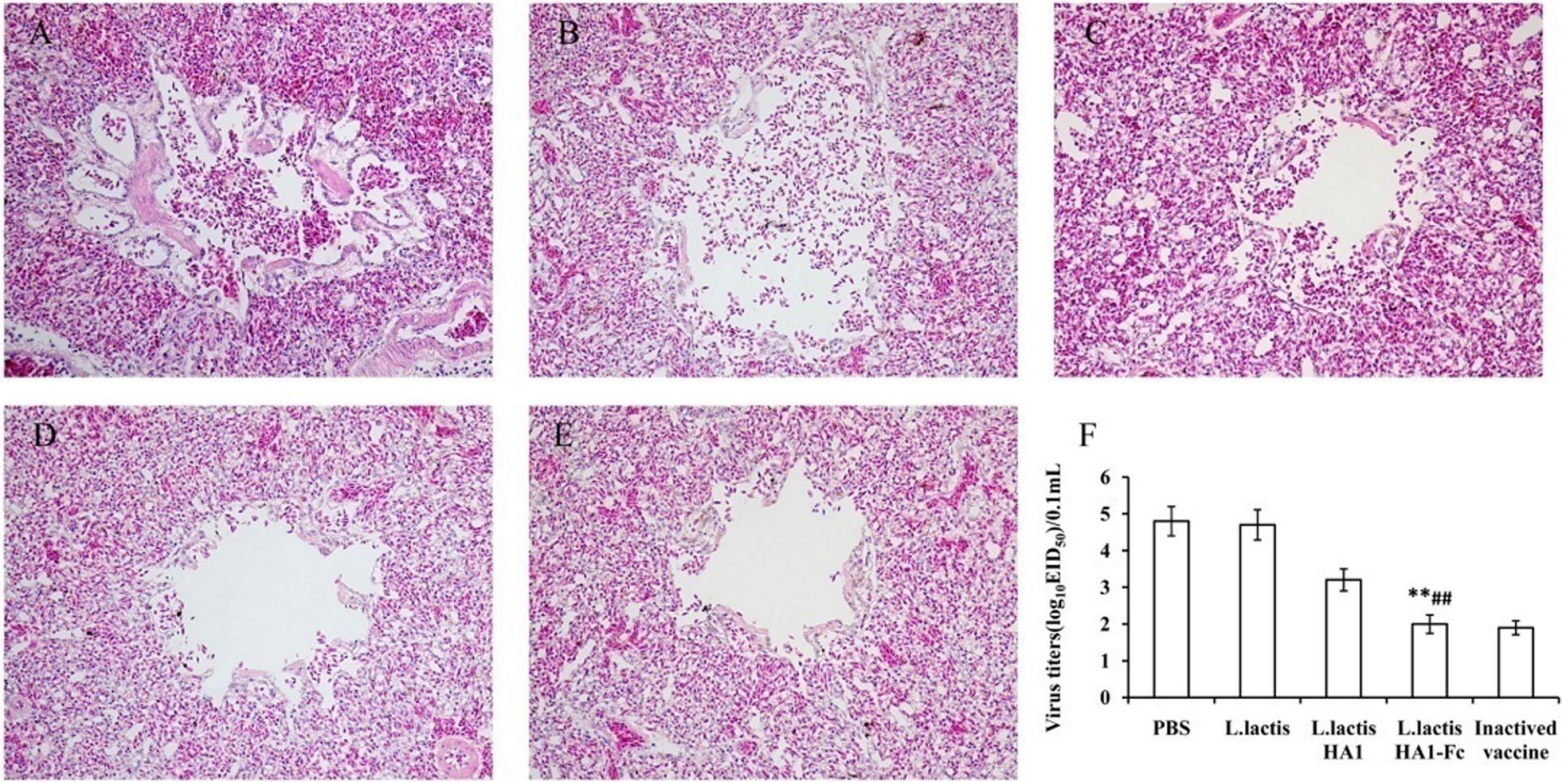
Protective effect of *L. lactis* HA1-Fc on lung injury induced by H9N2 influenza virus infection. Seven days after challenge with H9N2 avian influenza virus, lung tissue of PBS group (**A**), *L. lactis* group (**B**), *L. lactis* HA1 group (**C**), *L. lactis* HA1-Fc group (**D**) and H9N2 inactivated vaccine (**E**) was evaluated by histopathological analysis. The sections were stained with H&E. Objective magnification, × 200 (**A**–**E**). (**F**) The virus titers of lung samples from vaccinated chickens were obtained at 7 days after challenge and infectivity was measured by EID^50^. The data were showed as means ± SD (n = 5). **P < 0.01 relative to PBS and *L. lactis*. ^##^*P* < 0.01 relative to *L. lactis* HA1

To further evaluate the protective effect of the *L. lactis* HA1-Fc, pathological changes in chicken from each group were assessed. As illustrated in Fig. 8A–E, chicken in PBS group and *L. lactis* group showed significantly pathological changes, including increased blood, and lymphatic cells; hyperemia; the lung chamber collapsed, and the pulmonary epithelial cells detached.

By contrast, there were only minor pathological changes in the *L. lactis* HA1-Fc group and the inactivated vaccine group. Moreover, the pathological changes in *L. lactis* HA1-Fc group were significantly lighter than those in *L. lactis* HA1 group. The results confirmed that oral-administration *L. lactis* HA1-Fc could provide protection against the H9N2 AIV virus via reducing pulmonary pathology.

### Lung tissue virus titers assays

To evaluate the protective effect of recombinant *L. lactis* HA1-Fc strain on the H9N2 subtype AIV, lung tissue virus titers of each group were detected. The results showed that the lung virus titer in *L. lactis* HA1-Fc group was obviously lower than those in the PBS and *L. lactis* group (Fig. 8F). Moreover, compared with the *L. lactis* HA groups, the lung virus titer in *L. lactis* HA1-Fc group was also significantly reduced (*P* < 0.01). There was no significant difference in viral titer between *L. lactis* HA1-Fc group and inactivated vaccines group.

## Discussion

Avian influenza viruses (AIVs) have brought great threat to the poultry industry. At present, vaccination with inactivated vaccines for all poultry was the most common strategy for prevention and control of avian influenza. However, the immunogenicity of current AIV inactivated vaccines that mainly induce humoral immunity was limited. H9N2 influenza virus is mainly transmitted through the respiratory mucosal pathway, so mucosal immunity is considered to play a good role in controlling avian influenza infection. In this study, we fused IgY Fc and HA protective antigen genes of H9N2 AIV, thus constructing the recombinant *L. lactis* strain of HA1-Fc. The evaluation of the immunogenicity and protective efficacy showed that the recombinant *L. lactis* HA1-Fc strain induced substantial mucosal immunity and systemic immunity and had a good protective effect against H9N2 AIV challenge.

For a vaccine to be effective, the vaccine strain must have genetic and antigenic characteristics similar to those of the currently circulating field viruses. Therefore, it is pivotal to select the vaccine strain to prevent the spread of the virus. Since the mid-1990s, two different lineages of H9N2 AIV, called chicken A/Chicken/Beijing/1/94 and quail A/HongKong/G1/98, have been circulating in land poultry in Asia. The HA gene of A/pigeon/Hebei/02/2017 influenza virus was similar to the Ck/BJ/94-like lineage [41]. So we exploited a recombinant anti-H9N2 AIV vaccine based on this virus.

HA antibody is particularly important in the fight against infection and disease and is a crucial target for vaccine development [29]. It is reported that vaccines derived from HA gene as a protective antigen can induce subtype specific immunity and show efficacy against challenge with homologous virus [30, 42–44]. The HA1 domain contains many antigenic determinants that stimulate the production and binding of neutralizing antibodies [45]. Therefore, oral vaccine of recombinant *L. lactis* HA1-Fc was developed using HA1 as the target protein in this study, and our data showed that it could induce plentiful production of specific antibody. Although vaccination with the inactivated vaccine induced high HI antibody, no significant differences in HI antibody were detected in chickens immunized with inactivated vaccine and *L. lactis* HA1-Fc.

The linked IgY Fc mediated the interaction with macrophages and improved the expression of MHC-II molecules on macrophages. The upregulation of MHC-II promoted APC to present antigenic peptides to CD4+ T cells, which drive the activation of naïve T cells and obtain the help or regulation from CD4+ effector cells or regulatory T cells. So IgY Fc regions stimulates the activation of macrophages and increases the efficiency of antigen-processing, thereby improving the immune response induced by the antigen [20]. Therefore, the recombinant *L. lactis* HA1-Fc was constructed by connecting Fc to the target gene HA1 in this experiment, and the results showed that the oral vaccine of recombinant *L. lactis* HA1-Fc stimulated immune response, as evidenced by improved specific antibody and sIgA antibodies levels, increased lymphocyte proliferation, and increased IL-2, IL-4, and IFN-γ levels.

AIV utilize the host respiratory and gastrointestinal mucosal surfaces to initiate infections, therefore the ideal vaccine should stimulate mucosal and systemic immune reactions to restrict virus infection. So delivery of vaccine antigens through the mucosal surface would be an ideal approach to attain mucosal and possibly systemic immunity. However, mucosal epithelium is a natural barrier to a vaccine antigen transmission. On this basis, different approaches have been explored to solve this problem. FcRn can transport maternal IgG to the newborn via the placental path or intestinal [22], numerous reports have confirmed that targeting protective antigens to FcRn can increased humoral and cellular immune responses [23–25]. FcRY, the Fc receptor of avian IgY, was isolated from chicken yolk sac in 2004, and has been reported to expressed in many other tissues including intestinal tract [26]. Therefore, it is believed that FcRY has a similar transport function to mammalian FcRn, which can transport IgY in poultry intestinal tract [27, 28]. So we constructed a novel *L. lactis* HA1-Fc strain expressing a recombinant fusion protein consisting of the HA1 proteins and chicken IgY Fc fragment. Our results showed that the oral vaccine of recombinant *L. lactis* HA1-Fc showed increased mucosal immunity, as evidenced by improved sIgA levels in BALF and intestinal samples. The sIgA immunoglobulin is a critical component of the mucosal immune system. It can form a protective barrier by attaching to epithelial cells and it also can attach to newly synthesized viral proteins in infected cells, interfering with the assembly of virus particles. Notably, the sIgA level induced by recombinant *L. lactis* HA1-Fc was significantly higher than that induced by inactivated vaccine, so the recombinant *L. lactis* HA1-Fc oral vaccine induced higher mucosal immunity. Moreover, the probiotic effect of *L. lactis* promoted cellular response, and we also detected an up-regulation of IL-2, IL-4, and IFN-γ cytokines which promoted the activation and proliferation of the intestinal mucosal lymphoid B cells and induced the secretion of specific sIgA. They participated in the transmission of information and played an important role in the biological processes of the organisms. Our results show that immunization with *L. lactis* HA1-Fc can produce higher levels of IL-2, IL-4, and IFN-γ.

After virus challenge, our results showed that recombinant *L. lactis* HA1-Fc significantly inhibited weight loss, histopathological damage and inflammatory response in chickens, decreased virus titer in the lung, which was consistent with the immune protection of inactivated vaccine**.** So the recombinant *L. lactis* HA1-Fc can produced immune protection in chickens. Moreover, virus shedding was detected in oropharyngeal and cloacal swabs in the recombinant *L. lactis* HA1-Fc immunized chickenes at 2 days after challenge, and the titer was low and reduced rapidly to reach undetectable levels at 7 days after challenge. These results showed that the oral recombinant *L. lactis* HA1-Fc vaccine in this study could significantly inhibit the virus shedding. At present, inactivated H9N2 vaccines have demonstrated high efficacy in protecting against clinical disease, but variable results have also been observed in reducing the mortality and the level of viral shedding in chickens. It was reported that H9N2 AIV causes no death in chicken after intranasal challenge in control group [46] and no virus shedding in inactivated vaccine vaccination group [47]. However, other studies have shown that H9N2 AIV can cause death in chicken after intranasal challenge [48–50], and intranasal challenge of H9N2 virus resulted in different degrees of virus shedding in the inactivated vaccine group [46, 50–52], which was consistent with our results. Our previous studies demonstrated that intravenous rather than intranasal challenge of the H9N2 virus resulted in an extremely low degree of virus shedding, however, in order to mimic the natural route of infection, intranasal challenge of the H9N2 virus was chosen in this study. Indeed, our previous results are in line with findings that has been reported, whereby intravenous challenge of the H9N2 virus resulted in an extremely low degree of virus shedding [49].

## Conclusion

This study demonstrates that oral recombinant *L. lactis* HA1-Fc vaccine obtained good immune effect and can be used as a candidate vaccine for avian influenza prevention and control to compensate for the functional deficiency of inactivated vaccine.

## Data Availability

Data are available on reasonable request.

## Abbreviations

AIVs: Avian influenza viruses
*L. lactis*: *Lactococcus lactis*
HI: Hemagglutination inhibition
sIgA: Secretory IgA
BALF: Bronchoalveolar lavage fluid
HA: Hemagglutinin
